# *Cryptosporidium* spp. infections in Tunisia: Epidemiology and public health implications

**DOI:** 10.1002/vms3.71042

**Published:** 2026-06-29

**Authors:** Faten Bouaicha, Aurélie Chevillot, Sihem El Hamdi Mansour, Bruno Polack, Maxime Delsart, Karim Tarik Adjou

**Affiliations:** ^1^ Anses INRAE Ecole Nationale Vétérinaire d'Alfort UMR BIPAR Laboratoire de Santé Animale Maisons‐Alfort France; ^2^ Service de pathologie médicale du Bétail, University of Manouba Institution de la Recherche et de l'Enseignement Supérieur Agricoles Ecole Nationale de Médecine Vétérinaire de Sidi Thabet Sidi Thabet Tunisie; ^3^ Anses Ecole Nationale Vétérinaire d'Alfort Laboratoire de Santé Animale EPIMIM Maisons‐Alfort France

**Keywords:** *Cryptosporidium* spp, epidemiology, molecular prevalence, Tunisia, zoonoses

## Abstract

**Background:**

Cryptosporidiosis is an emerging worldwide zoonosis caused by an opportunistic protozoon, *Cryptosporidium* spp. In Tunisia, the lack of knowledge on the occurrence and genetic characteristics of *Cryptosporidium* spp. limits the understanding of its epidemiology, impact and transmission routes.

**Objectives:**

The present review aims to collect, compile and summarise data on the prevalence of *Cryptosporidium* spp. infections in humans, animals and the environment contamination in Tunisia.

**Methods:**

A systematic review was conducted across international databases using PRISMA 2020 Guidelines. Data from 15 selected studies were extracted and organised into thematic categories, including prevalence rates, risk factors, diagnostic methods, and population groups affected. A qualitative synthesis approach was used to analyse and compare findings across studies, highlighting trends, gaps, and public health implications.

**Results:**

The publications indicate that *Cryptosporidium* spp. infections are prevalent in both humans and animals, but the results remain confusing and may not reflect the real impact of the disease. In animals, only three studies were conducted in livestock and poultry. Only *Cryptosporidium parvum* (IIa and IId) was identified in the three studies from adult asymptomatic animals. Therefore, this finding may not reveal the true impact of the parasite on animal health. In humans, all studies were conducted in hospitals on adult and child patients diagnosed with either cancer or immunodeficiency. *Cryptosporidium parvum* and *Cryptosporidium hominis* were by far the predominant species suggesting potential zoonotic and anthroponotic transmission routes in humans. Of the two studies conducted on wastewater, five *Cryptosporidium species* (*C. parvum*, *C. hominis*, *Cryptosporidium meleagridis, Cryptosporidium muris, Cryptosporidium andersoni, Cryptosporidium ubiquitum*) and three genotypes were identified, highlighting the potential role of water as a potential route of *Cryptosporidium* spp. transmission.

**Conclusions:**

The current review give an overview of the circulating *Cryptosporidium* spp. species and genotypes but they remain insufficient to fully understand the transmission dynamics, zoonotic potential and animal welfare implications of *Cryptosporidium* spp. in Tunisia.

## Graphical abstract

Cryptosporidiosis, caused by *Cryptosporidium* spp., is a zoonotic disease affecting humans and animals worldwide. This review summarises available data on the prevalence and genetic diversity of *Cryptosporidium* spp. in Tunisia using published studies. Evidence indicates infections in humans, livestock, poultry, and wastewater, although data remain limited. *Cryptosporidium parvum* and *Cryptosporidium hominis* are the predominant species in humans, while only *C. parvum* has been identified in animals. Multiple species detected in wastewater highlight water as a potential transmission route, emphasising the need for further epidemiological studies.

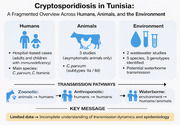

## 1 Introduction

*Cryptosporidium* spp. are intracellular protozoan parasites that infect a wide range of vertebrate hosts, including humans, domestic animals, and wildlife (Ryan et al. 2021a). This protozoan parasite remains a global Public Health concern, having been established as a leading cause of diarrhoea‐associated death in children and immunocompromised individuals in many developing countries in which the protozoan parasite is endemic (Sow et al. 2016; Kotloff et al. 2019a). The Global Enteric Multicentre Study has shown that *Cryptosporidium* spp. are a major cause of moderate‐to‐severe diarrhoea and mortality in young children in African and Asian countries (Delahoy et al. 2018; Kotloff et al. 2019b; Levine et al. 2020). It is also a frequent cause of water‐borne, and food‐borne, outbreaks of infection in many industrialised nations (Efstratiou et al. 2017; Zahedi and Ryan 2020). The transmission of *Cryptosporidium* spp. is primarily via a fecal‐oral route, occurring through the ingestion of environmentally resistant oocysts present in contaminated water, food, or via direct contact with infected hosts. Accessory contamination can also occur through inhalation of oocysts (Sponseller et al. 2014 Jul). To date, about 47 species of *Cryptosporidium* and more than 120 genotypes have been identified (Ryan et al. 2021b; Zhao et al. 2023), including *C. hominis* and *C. parvum*, which represent the 95% of cryptosporidiosis cases in humans (Lebbad et al. 2021).

In Tunisia, cattle, sheep and goat farming are the primary source of local food production, mainly meat and milk. They are also the main source of revenue for livestock farmers. However, Tunisian livestock is affected by a variety of parasitic infections, such as gastrointestinal parasites (Rouatbi et al. 2020; Hammami et al. 2024) or tick‐borne parasites (Khamassi Khbou et al. 2021). Cryptosporidiosis in neonatal calves, lambs and goat kids remains poorly documented. Only a few studies have been conducted namely in neonatal calves where *C. parvum* has been reported with a prevalence as high as 86.7% in a dairy farm in the Sfax region (Soltane et al. 2007a). However, genotyping was not conducted in this early report, limiting further interpretation of its zoonotic potential.

Environmental studies remain scarce, though preliminary investigations suggest the presence of oocysts in surface and drinking water sources, raising concerns about waterborne transmission pathways.

This review aims to provide a comprehensive summary of the current state of knowledge regarding *Cryptosporidium* spp. infection in Tunisia, by focusing on its prevalence, species distribution, subtypes, and potential transmission dynamics across human, animal, and environmental interfaces. By critically analysing the available data, we seek to identify gaps in surveillance, molecular characterisation, and public health policy, thereby guiding future research and control strategies.

## 2 Materials and Methods

A comprehensive literature search was conducted across five databases, PubMed, Google Scholar, ResearchGate, ScienceDirect and Cabi, to identify relevant publications. Articles published in local journals were also used after a ResearchGate search.

**FIGURE 1:**
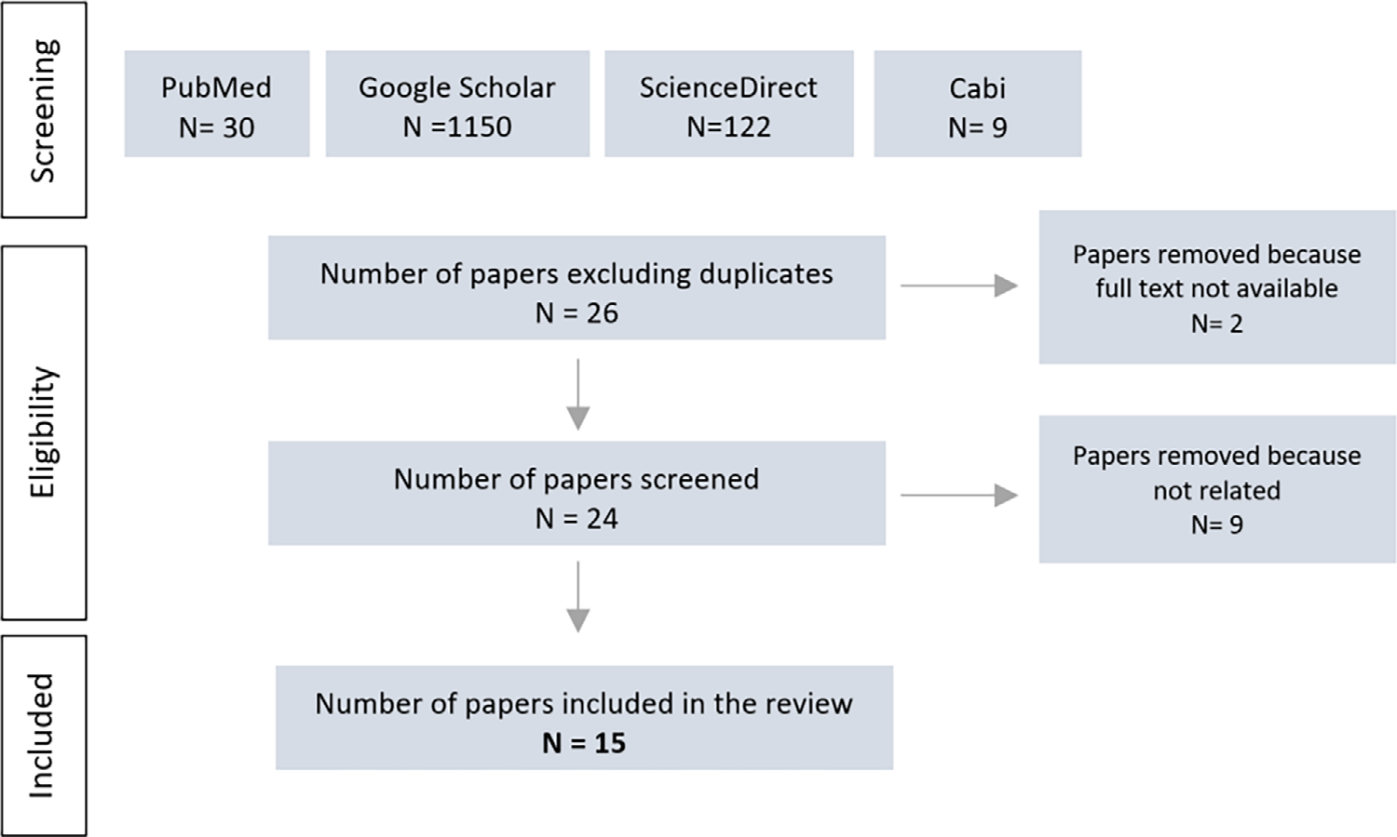
PRISMA diagram.

The search strategy was based on the use of specific keywords and their combinations, including ‘*Cryptosporidium*’, ‘cryptosporidiosis’, and ‘Tunisia’, focusing on studies conducted in humans, animals, and on environmental samples in Tunisia (Figure 1).

Therefore, using a PRISMA 2020 guidelines, only studies with an accessible full text that reported the detection, prevalence, and/or typing of *Cryptosporidium* spp. using molecular and/or microscopic methods in humans, animals, and environmental samples were included. Regional and systematic reviews were excluded.

A total of 15 peer‐reviewed research articles studying the infection by *Cryptosporidium* spp. in humans (*n* = 10) (Table 1), animals (*n* = 3) (Table 2) and detection in the environment (*n* = 2) (Table 3) were included in this review.

**TABLE 1:** Human *Cryptosporidium* spp. infection prevalence reported in Tunisia.

						Prevalence % (positive/examined samples)					
District	Study period	Population	Associated symptoms	Matrix	Diagnosis method	*Cryptosporidium* spp.	*C. hominis*	*C. meleagridis*	*C. parvum*	subtype	Reference
Bizerte	April–October 2007	Children < 5 years (*n* = 403)	Diarrhoea (*n* = 52)	Fecal samples	MZN / rRNA 18S, GP60 RFLP Sequencing			0.7% (3/403)	4/403 (0.09%)	IIaA15G2R1 (*n* = 2) IIdA16G1 (*n* = 2)	(Rahmouni et al. 2014)
Tunis	2006–2009	Children with primary immunodeficiencies	Chronic diarrhoea	Fecal samples	MZN, rRNA 18S, RFLP		3/5	1/5	1/5	NR	(Ben Abda et al. 2011)
Tunisian hospitals	2010–2015	HIV + (*n* = 174) Immunocompetent children (*n* = 260) Myeloma (*n* = 54) Immunocompromised children (*n* = 23) Colorectal cancer (*n* = 15)	Diarrhoea or vomiting	Fecal samples	Weber‐Green modified trichrome staining PCR RFLP 18S rRNA, sequencing		8/174 (4.5%) 4/260 (1.5%) 3/54 (5.5%) 3/23 (13%) 2/15 (13.3%)	2/174 (1.1%) 0 0 0 0	12/174(6.8%) 3/260 (1.1%) 0 1/23 (4.3%) 3/15 (20%)	***C. hominis*** IaA26G1R1 ***C. parvum*** IIaA14G2R1 IIaA15G2R1 IIaA16G2R1 IIaA20G1R1 IIdA15G2R1 IIdA16G2R1 IIdA19G2R1 ***C. meleagridis*** IIIbA26G1R1	(Essid et al. 2018)
Tunis	2010–2015	HIV patients (*n* = 174) Immunocompromised children (*n* = 23) Adults with myeloma (*n* = 54) Children < 5 years (*n* = 260)		Fecal samples	PCR, RFLP, sequencing		8/174 (4.59%) 3/23 (13%) 3/54 (5.5%) 4/260 (1.5%)			***C. hominis*** IaA26G1	(Essid et al. 2017)
Tunis	2006–2014	Patients with colorectal cancer (*n* = 39)		Intestinal biopsies	PCR‐HRM (DHFR gene)		0	0	5/39 (13%)	NR	(Jelassi et al. 2024)
Sousse	1991–2002	Immunocompetent children (*n* = 34,020)	Diarrhoea	Fecal samples	MZN		180/34,020 (0.32%)				(Fathallah et al. 2004)
Sfax	1997–2006	Children and adults (*n* = 30,573)		Fecal samples	MZN		17/30,573 (0.05%)				(Cheikhrouhou et al. 2009)

Abbreviations: HRM: high‐resolution melt; MZN: modified Ziehl‐Neelsen; NR: not reported.

**TABLE 2:** Farm animal *Cryptosporidium* spp. infection prevalence reported in Tunisia.

						Prevalence % (positive/examined sample)					
District	Study period	Animal species	Age	Associated symptoms	Diagnostics methods	*Cryptosporidium* spp.	*C. hominis*	*C. meleagridis*	*C. parvum*	subtype	Reference
Bizerte Joumine	April–October 2007	Calves (*n* = 70)	< 5 months	Diarrhoea (*n* = 52)	MZN 18S nested PCR RFLP Gp60 Sequencing				21.4 (15/70)	IIaA15G2R1 (*n* = 13) IIdA16G1 (*n* = 2)	(Rahmouni et al. 2014)
Sfax	2000	Calves (*n* = 30)	< 1 month	Diarrhoea	MZN Nested PCR Sequencing				86.7 (26/30)	NR	(Soltane et al. 2007a)
Tunis Nabeul Le Kef Siliana Sidi Bouzid Kairouan Médenine	2003–2004	Lambs (*n* = 30)	< 3 months		MZN Nested PCR Sequencing	16.7 (5/30)				NR	(Soltane et al. 2007b)
		Sheep (*n* = 59)	> 1 year			8.5 (5/59)					
		Goats (*n* = 184)	1–7 years			0					
		Horses (*n* = 190)	1–3 years			0					
		Rabbits (*n* = 178)	1–2 months			0					
		Camels (*n* = 110)	3–8 months			0					
		Chickens (*n* = 200)	0–56 days			(4.5) 9/200					
		Turkeys (*n* = 50)	NR			0					

Abbreviations: MZN: modified Ziehl‐Neelsen; NR: not reported.

**TABLE 3:** Environmental *Cryptosporidium* spp. prevalence reported in Tunisia.

	Positive samples/samples examined									
District	Plants	Raw wastewater	Treated wastewater	Sludge	Technique	Target gene	Species identified	Cryptosporidium oocyst/L	Genotype/subtype	Reference
Tunis	5/6	6/7	0/8	1/5	PCR – RFLP gp6	SSU rRNA	*C. parvum* *C. hominis* *C. muris* *C. andersoni*	1–21	***C. hominis*** IaA27R3 IdA15G1 ***C. parvum*** IIaA21R1 IIcA5G3	(Khouja et al. 2010)
Nationwide	16/18	42/110	8/110	5/12	PCR gp60	SSU rRNA	*C. parvum* *C. muris* *C. andersoni* *C. hominis* *C. ubiquitum* rat genotype, unknown *Cryptosporidium*, *C. meleagridis*, and avian genotype II (*C. ornithophilous*)	NR	*C. hominis* **Ia, Id** *C. parvum* **IIa, IIc**	(Ayed et al. 2012)

Abbreviation: NR: not reported.

## 3 Results

### 3.1 ***Cryptosporidium*** spp. **Infections in Humans**

**FIGURE 2:**
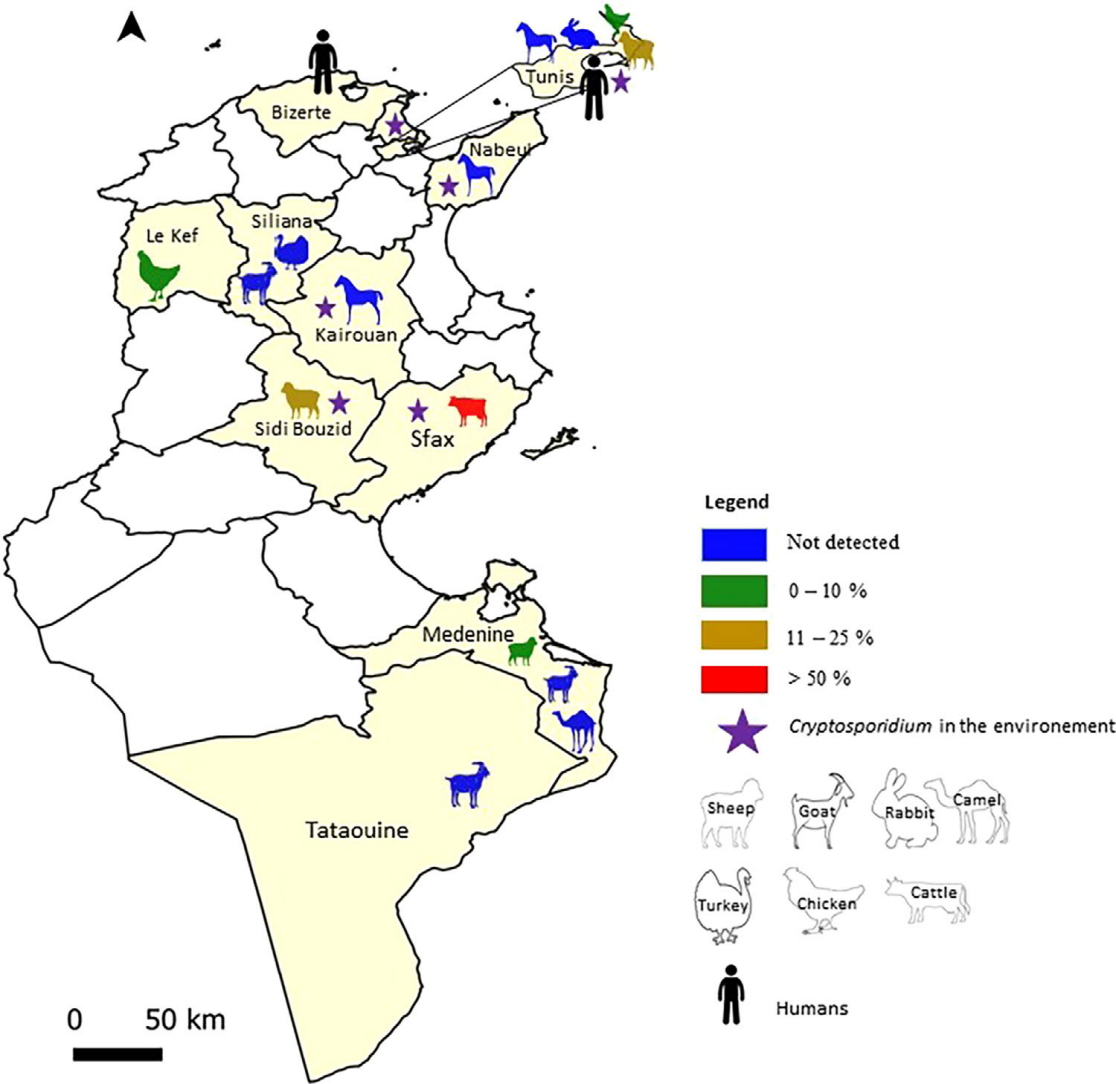
Map of Tunisia highlighting the main regions where *Cryptosporidium* spp. were detected (QGIS).

Ten surveys were conducted to investigate the epidemiology of *Cryptosporidium* spp. infection in humans in Tunisia (Table 1, Figure 2).

In all the studies, the target population was either adult patients with health issues (HIV, myeloma and colorectal cancers) and/or children less than 5 years old with either diarrhoea or an immune‐compromised system (Essid et al. 2008; Kourda et al. 2008; Cheikhrouhou et al. 2009; Essid et al. 2018). The infection prevalence varied from 0.05% to 20% with a higher prevalence in immuno‐compromised children. A range of clinical manifestations was observed for both asymptomatic and symptomatic patients. Symptoms included diarrhoea, abdominal pain, vomiting, anorexia, and weight loss.

*Cryptosporidium* species identified included *C. parvum*, *C. hominis*, and *C. meleagridis*. Among the ten studies reviewed, only three conducted subtype characterisations of the identified species (Essid et al. 2017, Essid et al. 2018; Rahmouni et al. 2014). Analysis of the gp60 glycoprotein revealed the presence of two *C. parvum* subtype families: IIa and IId. The IIaA16G2R1 subtype, which is commonly reported worldwide in both calves and humans, underscores the potential zoonotic role of calves as reservoirs. In this review, it was identified in two studies involving patients from both rural and urban areas (Essid et al. 2018; Rahmouni et al. 2014).

Only one subtype was identified for both *C. hominis* and *C. meleagridis*, IaA26G1R1 and IIIbA26G1R1 respectively (Essid et al. 2018; Essid et al. 2017).

Most studies investigating the epidemiology of *Cryptosporidium* spp. infection in humans have focused primarily on clinical aspects, such as symptoms, diagnostic methods, and the relationship between immunosuppression and clinical manifestations. However, risk factors and patterns of contamination have not been thoroughly examined.

### 3.2 ***Cryptosporidium*** spp. **Infections in Animals**

#### 3.2.1 Cattle

Only two studies investigated the presence of *Cryptosporidium* spp. in calves (Soltane et al. 2007a; Rahmouni et al. 2014). The first study was a longitudinal survey aimed at determining the prevalence of *Cryptosporidium* spp. infection on a dairy farm in the Sfax region (Soltane et al. 2007a). A total of 480 fecal samples were collected from 30 calves under 1 month of age. All samples were analysed for *Cryptosporidium* spp. oocysts using microscopic examination of smears stained by the modified Ziehl‐Neelsen (MZN) technique. The parasite was detected in 26 calves (86.7%). Molecular characterisation revealed that the isolates were *C. parvum*, although genotyping was not performed. This study was the first to identify *C. parvum* in calves in Tunisia.

The second investigation aimed to study the diversity of the glycoprotein 60 in *C. parvum* in both humans and cattle in two rural regions of Northern Tunisia. Among seventy fecal samples from pre‐weaned calves, 15 were shown to be positive by the MZN method. *Cryptosporidium parvum* was the only species identified. Genotype analysis revealed that IIaA15G2R1 and IIdA16G1 were the predominant subtypes (Rahmouni et al. 2014) (Table 2, Figure 2).

#### 3.2.2 Sheep and Goats

*Cryptosporidium* spp. were first detected in sheep from the Sidi Bouzid and Medenine districts. Ten fecal samples from 89 sheep yielded a positive result (25.2%), which were not genotyped. The infected sheep were apparently healthy at the moment of sampling. The infection prevalence was higher in lambs under 3 months (16.7%; 5/30 samples) compared to older sheep (8.5%; 5/59 samples) (Soltane et al. 2007b).

#### 3.2.3 Poultry

Over 250 chicken and turkey fecal samples were examined by MZN. Of the 200 chicken samples examined only 9 were observed to be positive for *Cryptosporidium* spp. by MZN staining (Soltane et al. 2007b). The parasite was not detected in turkeys.

#### 3.2.4 Camels

One study investigated the contamination of camels by *Cryptosporidium* spp. 110 healthy dromedaries aged between 3 to 8 months were sampled from the Ben Guerdan district. Fecal samples were examined by MZN stained and no parasites were observed (Soltane et al. 2007b).

*Cryptosporidium* spp. were not detected in horses aged between 1 to 3 years (*n* = 190) and in rabbits (*n* = 178) aged between 3 and 8 months using the MZN staining technique (Soltane et al. 2007b).

### 3.3 *Cryptosporidium* spp. Infections in the Environment

Two studies investigated the presence of *Cryptosporidium* spp. in wastewater and sludge samples from different sewage treatment plants located throughout Tunisia (Khouja et al. 2010; Ayed et al. 2012) (Table 3).

According to Khouja et al. (2010), of the 20 samples collected from six sewage treatment plants, *Cryptosporidium* spp. oocysts were detected in raw wastewater (5/7 samples), treated wastewater (1/8 samples) and sludge samples (4/5 samples). The contamination level was estimated by immunofluorescent microscopy and ranged between 1 and 21 oocysts/L. The contamination level was highest in raw wastewater.

In 2012, Ayed et al. (2012) evaluated the occurrence and geographical distribution of several zoonotic parasites including, *Cryptosporidium* spp., in raw wastewater (*n* = 110), treated wastewater (*n* = 110) and sludge (*n* = 12) collected monthly between 2005 and 2008 from 18 wastewater treatment plants. The results of the survey recorded a high prevalence and genetic diversity of pathogenic protists in treated and raw wastewater in Tunisia, including *Cryptosporidium* spp. Indeed, more than 6 *Cryptosporidium* species were detected with various subtypes. These findings suggest that the various sewage treatment processes adopted in Tunisia are insufficient in removing these parasites.

## 4 Discussion

Intestinal parasitic infections are of increasing importance worldwide, especially in developing countries. They affect both humans and animals and are highly resistant in the environment. *Cryptosporidium* spp., an intracellular protozoan parasite, have been recognised as an important cause of moderate to severe diarrhoea in children less than 5 years old (Kotloff et al. 2019a; Platts‐Mills et al. 2018). However, the potential economic impact and welfare implications of *Cryptosporidium* spp. infection remain poorly documented in North African.

This review provides a valuable summary of the occurrence of *Cryptosporidium* spp. in humans, animals and the environment in Tunisia. Based on published studies, *C. parvum*, *C. hominis*, and *C. meleagridis* are the species identified as being responsible for human cryptosporidiosis in Tunisia. Reported infection prevalence ranged from 0.09% (4/403 samples) for *C. parvum* in children under five years of age to 20% (3/15 samples) in patients with colorectal cancer. *Cryptosporidium parvum* was identified in four out of five studies, while *C. hominis* and *C. meleagridis* were detected in three of five studies, indicating similar frequencies. These findings are similar to the known distribution of *Cryptosporidium* species in Europe, where *C. parvum* and *C. hominis* are the most commonly reported species in humans (Chalmers et al. 2005; Leoni et al. 2006; Xiao 2010). This is notable, as both species are frequently associated with waterborne and foodborne outbreaks of human cryptosporidiosis (Xiao and Feng 2008).

Interestingly, *C. meleagridis* appears to be as prevalent as *C. hominis* in children from urban areas of Tunisia. A similar pattern has been reported in other regions of the world, such as Bangkok, where zoonotic species were found to be as prevalent as anthroponotic ones among HIV‐infected patients (Gatei et al. 2002). However, in the Tunisian context, these findings remain inconsistent and inconclusive, highlighting the need for further research to assess species‐specific infection risks, especially given that transmission routes (zoonotic vs. anthroponotic) vary between species.

All the reviewed studies targeted either healthy children or individuals with cancer or immunodeficiency and were mostly conducted in hospitals. The focus in these areas underscores the significant importance of *Cryptosporidium* spp. as a serious threat in vulnerable populations, emphasising the need for enhanced surveillance systems to improve early detection and response.

Molecular characterisation of *Cryptosporidium* species and subtypes plays a crucial role in identifying infection sources, particularly during outbreaks, and is essential for developing control and prevention strategies (Fan et al. 2019; Yang et al. 2021). In Tunisia, species identification and genotyping were conducted in some studies focusing on human infection. Molecular analysis of *C. hominis* at the subtype level, based on gp60 gene polymorphism, revealed that all isolates belonged to a single subtype family (Ia), with IaA26G1R1 being predominant. The detection of a single subtype family across different individuals suggests endemic transmission and highlights the role of anthroponotic transmission. Moreover, the consistent presence of the IaA26G1R1 subtype among HIV‐infected patients attending the same hospital suggests a possible nosocomial transmission (Vincent et al. 2009; Weber and Rutala 2001). This transmission route is well‐documented in intensive care units for various bacterial and fungal pathogens (Lemiech‐Mirowska et al. 2021). More recently, increasing attention has been given to the role of parasitic organisms, including *Cryptosporidium* spp., in nosocomial infections (Kosik‐Bogacka et al. 2024; Fürnkranz and Walochnik 2021; Brunet et al. 2016).

The data available from Tunisia regarding cryptosporidiosis in humans are valuable but incomplete making an in depth understanding of *Cryptosporidium* spp. transmission dynamics in the different regions of the country difficult.

Only three studies have assessed the prevalence of *Cryptosporidium* spp. infections in farm animals in Tunisia. These studies primarily report infections in livestock but are subject to several limitations.

Firstly, the study population selected was not optimal, because most of the animals sampled were adults (over one year of age) and asymptomatic, factors associated with a lower probability of detecting oocysts. In contrast, two studies conducted by Soltane et al. (2007b) and Rahmouni et al. (2014) focused on younger animals, specifically calves and lambs under five months of age, and reported significantly higher infection prevalences of 86.7% and 11.2%, respectively. These findings are consistent with existing evidence indicating that *Cryptosporidium* spp. infections are more frequent in young animals and may be significantly underestimated when surveillance is restricted to adult livestock. Furthermore, the available studies did not target farms with known cryptosporidiosis or a history of cryptosporidiosis. Consequently, the published data do not accurately reflect the true prevalence of *Cryptosporidium* spp. in neonatal diarrhoea among farm animals in Tunisia, despite the well‐documented role of this parasite in such cases worldwide (Jang et al. 2021; Li et al. 2019; Mammeri et al. 2019). The lack of locally relevant epidemiological data hampers efforts to assess the pathogen's impact on farm animal health and hinders the implementation of appropriate evidence‐based control programs.

Secondly, in most studies, diagnosis relied on the morphological identification of oocysts in fecal samples using microscopy techniques, especially the modified Ziehl‐Neelsen technique. While this method is accessible and inexpensive, it lacks the sensitivity and specificity of molecular techniques, potentially leading to underestimation of the true prevalence and diversity of *Cryptosporidium* species (Chalmers et al. 2011; Omoruyi et al. 2014). In fact, *C. parvum* was the only species identified in the three studies conducted in livestock animals in Tunisia. Further studies are needed to confirm these results.

This diagnostic limitation not only hinders effective surveillance but also impairs understanding of the zoonotic potential of these infections. Given the close interaction between humans and farm animals in many rural and peri‐urban areas of Tunisia, there is a pressing need to adopt molecular tools for accurate species and subtype identification. Such developments would improve disease monitoring and help clarify the role of animal reservoirs in human cryptosporidiosis.

In contrast, *Cryptosporidium* spp. infections are better characterised in Algeria, a neighbouring country that shares a 1020 km border with Tunisia and where there is significant movement of livestock between the countries. Ouakli et al. (2018) reported a molecular prevalence of *Cryptosporidium* spp. infection up to 52.2% (240/460 samples) in cattle aged between 2 days and 18 months, sampled from 10 farms distributed across the country. Moreover, another study identified the circulating species and the zoonotic subtypes in *C. parvum* IIa and IId in 41 calves (Sahraoui et al. 2023).

In conclusion, the currently available data on *Cryptosporidium* spp. infection in animals from Tunisia cannot be considered representative of the national situation. Community‐based studies employing molecular tools and a well‐designed sampling strategy are needed to: (i) determine the prevalence of cryptosporidiosis in the context of neonatal diarrhoea across different farms; (ii) identify circulating *Cryptosporidium* species to better understand contamination routes and assess zoonotic risk; and (iii) establish robust epidemiological surveillance to monitor parasite circulation.

Only two studies reported the contamination of the environment by *Cryptosporidium* spp. Findings from the two studies indicate a significant prevalence and genetic diversity of *Cryptosporidium* spp. in wastewater samples from various sewage treatment plants in Tunisia. The assessment of environmental contamination by *Cryptosporidium* spp. oocysts is limited to wastewater, without sufficient consideration of other exposure pathways such as drinking or irrigation water. The results confirmed the presence of *Cryptosporidium* spp. at varying concentrations and notable genetic diversity. Considering the role of wastewater‐based epidemiology as an epidemiological tool for monitoring parasites (Puchades‐Colera et al. 2025), the implication of these findings is not fully developed. In fact, due to Tunisia's limited water resources, treated wastewater has increasingly been used as an alternative source for domestic, industrial, and agricultural purposes. By 2007, approximately 30% of treated wastewater was used to irrigate nearly 9000 hectares of farmland (Office Nationale d'Assainissement 2012). Consequently, the government has established maximum limits for various chemicals, bacteriological contaminants, and parasite eggs. However, these regulations do not include protozoa such as *Cryptosporidium* spp. and *Giardia duodenalis*, which are frequently responsible for waterborne diarrhoea outbreaks in many countries (Bourli et al. 2024; Watier‐Grillot et al. 2022; Chalmers et al. 2021).

Results from the studies on the environment, suggest that current wastewater treatment methods may be insufficient to effectively eliminate *Cryptosporidium* spp. oocysts, highlighting a possible public health concern related to wastewater reuse. However, the actual risk of exposure and transmission associated with this practice remains uncertain and has not been adequately quantified. A more rigorous evaluation of health risks to humans and animals would require: (i) systematic monitoring of *Cryptosporidium* spp. oocysts before and after wastewater treatment; (ii) analysis and quantitative assessment of *Cryptosporidium* spp. oocysts in drinking water; and (iii) species‐level identification to better understand contamination sources and transmission pathways.

## 5 Conclusions and Perspectives

This review compiles existing data on the occurrence of *Cryptosporidium* spp. infection in Tunisia, providing valuable insights while also highlighting several important gaps in our knowledge.

The currently data available from Tunisia remain insufficient to fully elucidate the real impact and the transmission dynamics of *Cryptosporidium* spp. in both animals and humans. There is a significant gap in understanding the role of *Cryptosporidium* spp. in neonatal diarrhoea in farm animals. Moreover, existing studies are restricted to geographical zones therefore limiting the ability to draw nationwide epidemiological conclusions.

Additional investigations across diverse regions are therefore necessary to obtain a more comprehensive understanding of the parasite's epidemiological distribution. Future research should prioritise: (i) expanded molecular studies to determine the prevalence of cryptosporidiosis in farm animals and to identify circulation species and subtypes; (ii) detailed assessment of human risk factors, particularly those related to lifestyle, occupational exposure, and hygiene practices; (iii) identification of transmission routes, especially in rural populations with frequent contact with livestock; and (iv) development of targeted prevention and control strategies in high‐risk settings.

Accordingly, robust epidemiological surveillance within a One Health framework is essential to accurately estimate infection prevalence and to develop effective, evidence‐based intervention strategies.

## Author Contributions

**Faten Bouaicha**: conceptualisation, investigation, writing – original draft, methodology, writing – review & editing. **Aurélie Chevillot**: writing – original draft, writing – review & editing. **Sihem El Hamdi**: writing – original draft, writing – review & editing. **Bruno Polack**: writing – original draft, writing – review & editing, supervision. **Maxime Delsart**: writing – review & editing, validation, methodology. **Karim Tarik Adjou**: conceptualisation, writing – original draft, writing – review & editing, validation, methodology, supervision

## Funding

The authors have nothing to report.

## Ethics Statement

This research did not require an ethical approval.

## Conflicts of Interest

The authors declare no conflicts of interest.

## Data Availability

Data sharing not applicable to this article as no datasets were generated or analysed during the current study.
